# Molecular Dynamics Investigation of Residual Stress and Surface Roughness of Cerium under Diamond Cutting

**DOI:** 10.3390/mi9080386

**Published:** 2018-08-03

**Authors:** Yao Li, Maobing Shuai, Junjie Zhang, Haibing Zheng, Tao Sun, Yang Yang

**Affiliations:** 1 Science and Technology on Surface Physics and Chemistry Laboratory, Mianyang 621908, China; bk7041638@sina.com (Y.L.); yangmax0901@163.com (Y.Y.); 2 Center for Precision Engineering, Harbin Institute of Technology, Harbin 150001, China; zheng_hai_bing@126.com (H.Z.); spm@hit.edu.cn (T.S.)

**Keywords:** cerium, diamond cutting, residual stress, roughness, molecular dynamics

## Abstract

Machined surface quality in terms of residual stress and surface roughness has an important influence on the performance of devices and components. In the present work, we elucidate the formation mechanisms of residual stress and surface roughness of single crystalline cerium under ultraprecision diamond cutting by means of molecular dynamics simulations. Influences of machining parameters, such as the rake angle of a cutting tool, depth of cut, and crystal orientation of the workpiece on the machined surface quality were also investigated. Simulation results revealed that dislocation activity and lattice distortion are the two parallel factors that govern the formation of both residual stress and surface roughness. It was found that both distributions of residual stress and surface roughness of machined surface are significantly affected by machining parameters. The optimum machining parameters for achieving high machined surface quality of cerium by diamond cutting are revealed.

## 1. Introduction

Cerium, as a lanthanide element with an atomic number of 58, is an important rare earth material. Cerium has been widely used in many industrial and civil fields, and has high practical value and research significance. Machined surface quality, in terms of residual stress and surface roughness, is one of the most important factors for improving the performance and life cycle of cerium-based components and parts. Thus, it is critical to improve machined surface quality of cerium by gaining fundamental understanding of formation mechanisms of residual stress and surface roughness, as well as their dependence on machining parameters. Ultraprecision diamond cutting is an important mechanical machining technique for achieving high machined surface quality [1,2,3]. 

Considerable work has been carried out to investigate the machined surface quality in diamond-cutting process. Tazehkandi et al. studied the cutting forces and surface roughness in turning of Inconel 738 and presented the optimum condition [4]. Yao et al. investigated the influence of cutting parameters on residual stress in cutting of TB6 [5]. Patrik et al. performed cutting experiments of quenched steel with changing of inclination angle and cutting parameters, and found that the main factors affecting the residual stress were the rake angle of the tool [6]. However, the literature review showed that either experimental or theoretical work about the diamond cutting of cerium has been rarely reported. The deformation behavior of cerium under diamond cutting is complex for its unique chemical, physical, and mechanical properties. Cerium has multiple lattice structures accompanied with rich paths of phase transformation [7,8,9,10,11]. It has been demonstrated that there are seven kinds of solid phases in cerium: *γ* (face centered-cubic, FCC), *α* (FCC), *β* (double hexagonal close packed, DHCP), *δ* (body centered-cubic, BCC), *α*’ (C-type orthorhombic or the *α*-U structure), *α*’’ (C2/m monoclinic) and *ε* (body centered tetragonal, BCT). The phase transformation from *γ*-Ce, which is stable under normal temperature and pressure, to *α*-Ce at 8 kbar and 295 K is accompanied with a large volume collapse of 20% [12,13,14]. The phase transformation-induced modification of the electronic structure and bonding configuration in cerium inevitably has a strong impact on its deformation behavior. In addition, cerium shows high plasticity, which may lead to undesired shaping flow under mechanical processing [15]. In particular, while diamond cutting is a highly coupled process between workpiece material and cutting tool, it is difficult to obtain high quality of machined surface of cerium due to the above special features of cerium. Previous work demonstrated that although dislocation slip is the dominant deformation mechanism of cerium under diamond cutting, there are trivial phase transformations from *γ*-Ce to *δ*-Ce occurred in both the machined surface and the formed chip [16,17].

While experimental investigation of a machining process is limited by the resolution of the machine tool and measuring appratus due to the ultrasmall materoval depth, theoretical investigation could provide microscopic dynamic details of an ongoing machining process. Given the discrete characteristics of material removal, conventional continuum mechanics theory cannot be applied to the analysis of the nanometric cutting process due to the size limitation. Molecular dynamics (MD) simulation has been demonstrated to be a powerful tool for studying various manufacturing processes at the nanometer scale, as it can reveal various phenomena that are out of reach by traditional approaches. In particular for mechanical machining processes, many researchers have adopted MD simulations to study a number of issues that are challenging for traditional numerical simulations or experimental approaches. Chavoshi et al. conducted an MD simulation of nanometric cutting on silicon in specific combinations of crystal orientation and cutting direction using a diamond tool [18]. Li et al. reported that the minimum wear depth of single crystalline Cu(111) under nanoscratching that is equivalent to the critical penetration depth at which plasticity initiates increases with probe radius [19]. In addition, previous studies also widely investigated the effect of machining parameters on machining processes, or the sensitivity of the established model in parameter selection [20,21]. However, there is limited work that investigated the formation mechanisms of residual stresses and surface roughness generated in nanomachining. Therefore, it is necessary to understand the formation mechanisms and distribution of residual stress and surface roughness in the diamond cutting with the aid of MD simulations.

Experimental and theoretical work has demonstrated that machining parameters have a strong impact on cutting processes. Xu et al. studied the influence of the hard particle on the surface generation, plastic deformation, and processing forces by means of MD simulations in nanocutting of aluminum [22]. Li et al. found that the plastic deformation for different scratching rates depends on the competition of scratching force, workpiece temperature, and tool–workpiece contacting time, which affect dislocation evolution [23]. Zhu et al. built a model to investigate the atomic force microscope (AFM)-based nanometric cutting process of copper using a diamond tool [24]. The effects of toolgeometry, cutting depth, cutting velocity, and bulk temperature were studied. To et al. experimentally studied diamond cutting of single-crystal aluminum with different crystal orientations [25]. It was found that dislocation density of different crystal plane is different, the highest is Ce(111) crystal plane, and the lowest is Ce(110) plane. The degree of difficulty of the dislocation movement will affect material plastic deformation resistance and further affect surface quality of materials. Ding et al. carried out diamond cutting of oxygen-free copper and found that internal crystal orientations will lead to the fluctuation of cutting force and uneven distribution of surface roughness [26]. Lucazeau and Abello adopted micro-Raman spectroscopy to study the residual stress field induced by microindentation by mapping the indented zones [27]. They found that amorphous silicon exists in the center of indentation and the amorphous silicon is partly recrystallized. Other work also used micro-Raman spectroscopy to study phase transition and residual stress in crystalline silicon induced by machining [28,29]. Romero et al. found that the adhesion during orthogonal cutting of a copper substrate could be reinforced by varying the tool rake angle or by choosing specific lattice orientations [30]. Besides, several modeling approaches have been developed to study residual stresses in machining. Tian et al. built a two-dimensional axisymmetric finite element model to predict the full-field residual stress in grounding of silicon wafers [31]. Winsner et al. developed a two-dimensional model to investigate the effect of temperature field on residual stress of aluminum alloy after high-speed cutting [32]. Although previous studies have provided valuable insights into the machining parameter dependence of cutting processes, there is no report on the influence of machining parameters on residual stress and surface roughness of cerium under diamond-cutting processes.

Therefore, in the present work, we perform MD simulations to elucidate the formation mechanisms of residual stress and surface roughness of cerium under diamond cutting process. Furthermore, the influences of rake angle of a cutting tool, crystallographic orientation of workpiece, and depth of cut (DOC) on the residual stress and surface roughness in diamond cutting of cerium are studied.

## 2. Simulation Method

**Figure 1:**
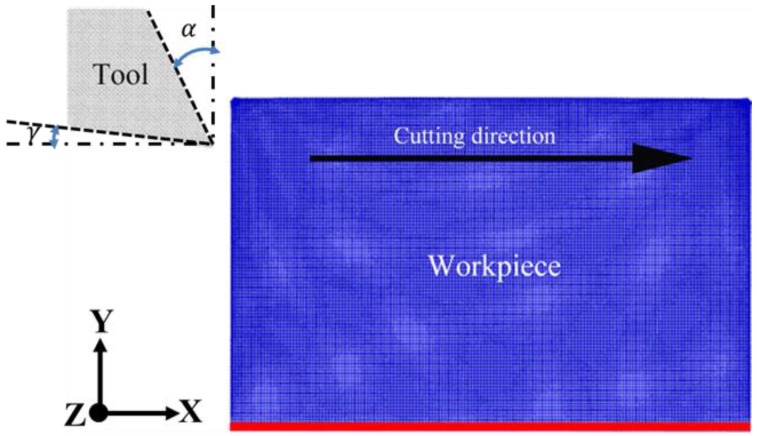
Molecular dynamics (MD) model of diamond cutting of cerium. *α* and *γ* are rake angle and clearance angle, respectively.

The MD model of diamond cutting consists of a rigid diamond tool and a single crystal cerium workpiece, as indicated in Figure 1. The cerium workpiece had a dimension of 41, 25, and 31 nm in the X, Y, and Z direction, respectively. To investigate the influence of crystal orientation, three cerium workpieces with Ce(100), Ce(110) and Ce(111) free surface in Y direction were considered. The diamond-cutting tool with a sharp edge had a relief angle *γ* of 9°. Seven rake angles α, as 10°, 20°, 30°, −10°, −20°, −30°, and 0°, were utilized to address the influence of rake angle. The Ce-Ce interaction between workpiece atoms and the Ce-C interaction between workpiece and diamond tool are described by the embedded atom method (EAM) [33] and Morse potential [16].

After the relaxation of the system at 300 K for 50 ps, the diamond tool cut the workpiece with a constant cutting speed of 100 m/s and a constant DOC until a predetermined travel distance of 60 nm was reached. To fully characterize the formation of residual stress and surface roughness, the cutting tool was then withdrawn after achieving the aforementioned travel distance. In this way, the machined surface went through full relaxation. All the MD simulations are based on LAMMPS developed by Sandia National Laboratory (PO Box 5800, Albuquerque, NM, USA) [34]. The Ovito was utilized to perform visualization of MD simulation of the machining process [35]. The detailed description of MD model of diamond cutting can be found elsewhere [16,17].

**Figure 2:**
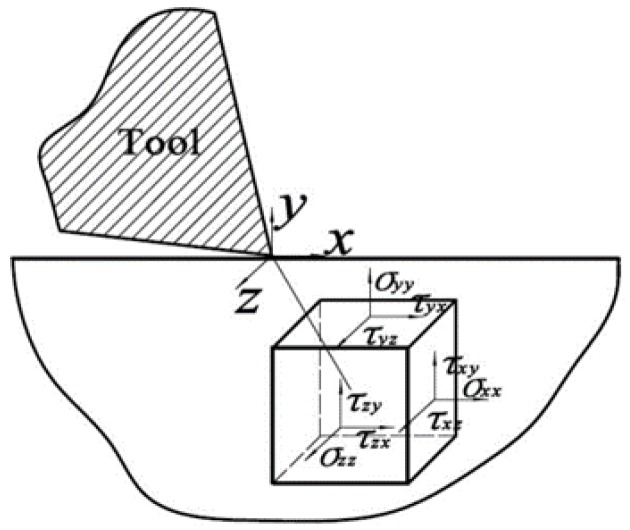
Schematic diagram of stress components in diamond cutting.

The cutting force acting on the diamond tool in vector form can be expressed in Equation (1) as:

(1) $$F=\overset{N_{T}}{\underset{i=1}{\sum }}\underset{j}{\sum }\frac{\partial U\left(r_{ij}\right)}{\partial r_{ij}}$$

where *F* is the cutting force, $N_{T}$ is the number of atoms in the cutting tool, *U* stands for the pairwise Morse potential, $r_{ij}$ is the distance between atom *i* and *j*. There are three components of machining force acting on the diamond tool, as cutting force along horizontal direction, normal force perpendicular to machined surface and lateral force along longitudinal direction, respectively. In addition, the six stress tensors, as $\sigma_{xx}$, $\sigma_{yy}$, $\sigma_{zz}$, $\sigma_{xy}$, $\sigma_{xz}$, and $\sigma_{yz}$ shown in Figure 2, for each atom can be derived from viral stress shown in Equation (2):

(2) $$\chi =\frac{1}{\Omega }\overset{N}{\underset{i}{\sum }}\left(m_{i}v_{i}⊗v_{i}+\frac{1}{2}\underset{i\neq j}{\sum }r_{ij}⊗f_{ij}\right)$$

where *χ* denotes the average viral stress with six components, Ω is the volume of the cutoff domain [23,36], *N* stands for the total number of atoms in the domain, and $m_{i}v_{i}$ is the momentum of the atom *i*, ⊗ is the tensor product of two vectors, $f_{ij}$ stands for the individual interaction force exerted on atom *i* by atom *j*. In addition, the equivalent Von Mises stress can be calculated in Equation (3):

(3) $$\sigma^{2}=\frac{1}{2}[{\left(\sigma_{xx}-\sigma_{yy}\right)}^{2}+{\left(\sigma_{yy}-\sigma_{zz}\right)}^{2}+{\left(\sigma_{zz}-\sigma_{xx}\right)}^{2}+6\left(\tau_{xy}^{2}+\tau_{yz}^{2}+\tau_{zx}^{2}\right)]$$

While the surface of single-crystal cerium workpieces can be considered to be uniform and regular, surface roughness characterization uses the surface contour arithmetic mean deviation $R_{a}$ as one of the evaluation parameters, which can be expressed in Equation (4) as:

(4) $$R_{a}=\frac{1}{l_{r}}\int_{0}^{l_{r}}\left|y_{i}\right|dx$$

**Figure 3:**
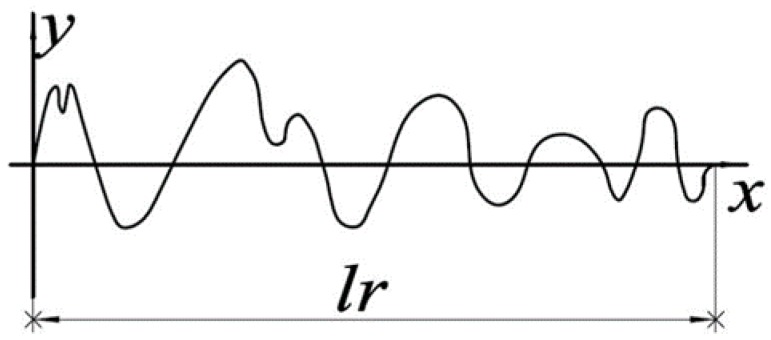
Schematic diagram of surface profile.

As shown in Figure 3, $y_{i}$ is the height of sampling point, $l_{r}$ is the sampling length. While cerium is a metallic material of high plasticity, there are grooves, holes, and other surface microtopography characteristics formed on the machined surface in the diamond cutting process. Therefore, local and random information of surface morphology needed to be expressed clearly. It should be noted that 3D surface roughness parameters that characterize the full surface morphology of the area would provide more comprehensive and accurate information than the current 2D surface roughness parameters. In this work, the more complex profile $R_{q}$ as another evaluation parameter is adopted, which is described in Equation (5) as:

(5) $$R_{q}=\sqrt{\frac{1}{l_{r}}\int_{0}^{l_{r}}y_{i}^{2}dx}$$

## 3. Results and Discussion

### 3.1. Formation Mechanisms of Residual Stress and Surface Roughness

**Figure 4:**
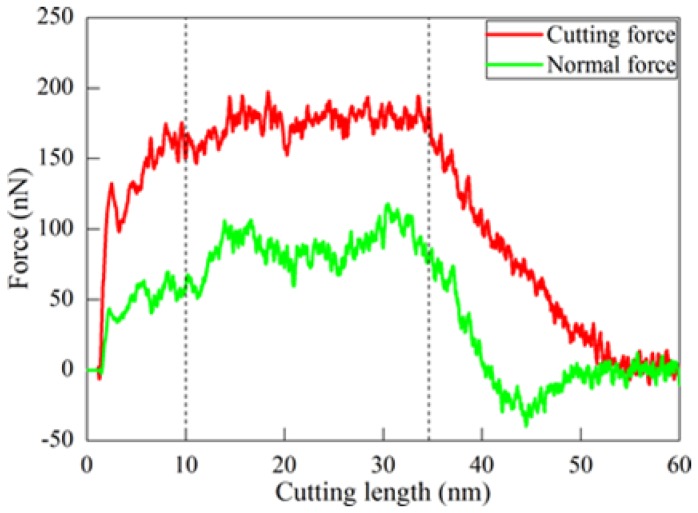
Variation of cutting force and normal force in cutting process of Ce(100).

The formation mechanisms of residual stress and surface roughness were first investigated by MD simulation of diamond cutting of Ce(100). The cutting tool with a rake angle of 0^o^ was utilized. The DOC is 20 Å. Figure 4 plots variations of cutting force and normal force with cutting length, indicating that the cutting process could be categorized into three phases as highlighted by the dash lines. The workpiece material first underwent pure elastic deformation, accompanied with a rapid increase of machining forces. After reaching a cutting length of 3 nm, plastic deformation occurred by dislocation emissions from the top surface and left surface. Consequently, machining forces dropped slightly. Upon further advance of diamond tool, the cutting process became stable, and machining forces fluctuated around stable values. Simultaneously, considerable dislocations nucleated from free surface and subsequently glided along slip planes under the stress applied by the diamond-cutting tool. After the diamond tool separated from the workpiece, machining forces decreased with increasing cutting length, accompanied with significant dislocation reaction and annihilation at free surface due to the release of surface stress. The detailed description about the microscopic deformation of materials and its correlation with macroscopic machining results can be found elsewhere [16,17].

**Figure 5:**
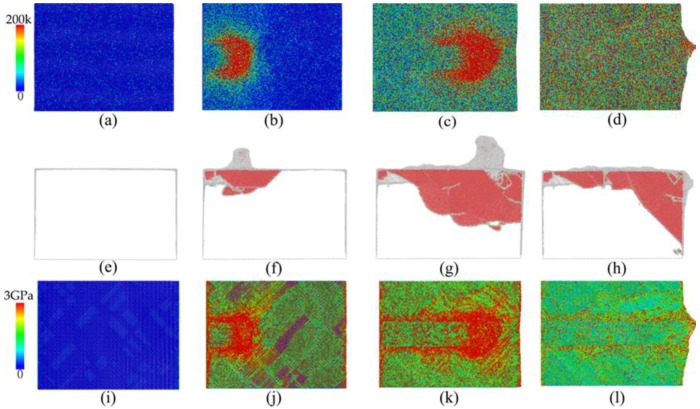
Evolution of cerium workpiece surface at different stages of the machining process. (**a**–**d**) present variations of temperature. (**e**–**h**) present instantaneous defect structures, atoms are colored according to calculated common neighbor analysis (CNA) values, and FCC atoms are not shown. (**i**–**l**) present variations of stress. The first, second, third and fourth row represent a cutting length of 0, 10, 30, and 60 nm, respectively.

**Figure 6:**
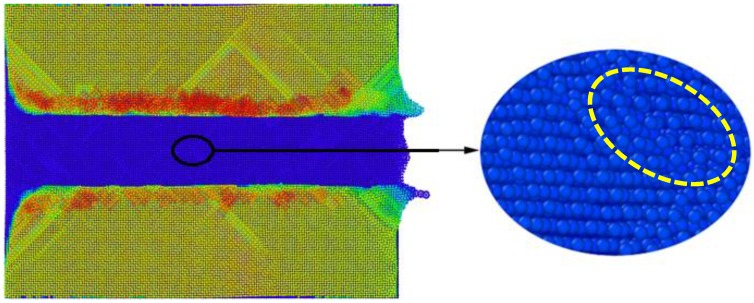
MD snapshot of machined surface morphology.

Due to the friction between the rake face of the cutting tool and displaced workpiece material, large amount of heat was produced in the cutting process, which led to a significant increase of temperature in the cutting zone. It is seen from Figure 5a–d that the generated heat distribution was not uniform. Correspondingly, Figure 5e–h presents instantaneous defect structures formed within the workpiece, which shows that the distribution of dislocations coincides well with the distribution of heat. In addition, the workpiece had serious local plastic deformation that led to the nonuniform change of the surface morphology from a flat surface to a rough one, as shown in Figure 6. In particular, the zoom view highlighted by the yellow ellipse in Figure 6 shows that the arrangements of local atoms on the machined surface became disordered, which was caused by the incompletely recovery of plastic strains. While a large number of dislocations were produced within the workpiece by plastic deformation, subsequent dislocation slipped and reaction events led to the formation of local stress concentration, as shown in Figure 5i–l. New energy was required for atoms in the defect zone of the workpiece material to maintain their balance. The vast majority of stored energy was consumed by material deformation to compensate for lattice distortion, which further increased the energy of the deformed crystals to reach thermodynamically unstable states. Furthermore, a spontaneous tendency to revert to the lowest stable state of free enthalpy, which is the origin of residual stress formation, occurred. A careful examination of atomic arrangements showed that lattice distortion was mainly localized in the topmost layers of the machined surface. Correspondingly, the residual stress of machined surface can be obtained by averaging atomic stress in the topmost layers. The calculated surface roughness $R_{a}$ and residual stress were 1.67 Å and 0.84 GPa, respectively. 

### 3.2. Effect of Rake Angle

**Figure 7:**
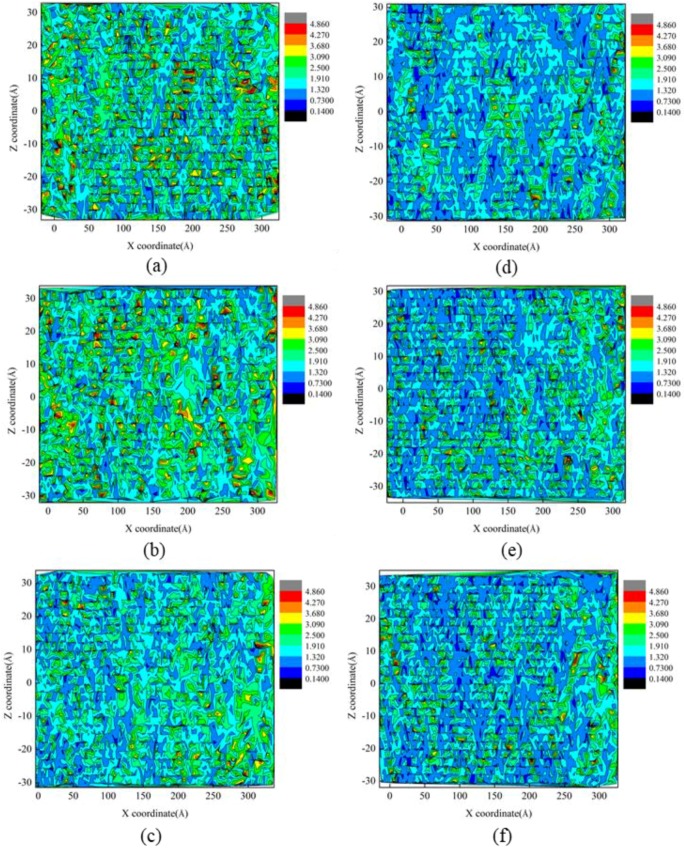
Residual stress of machined surface under different rake angles: (**a**) −30°, (**b**) −20°, (**c**) −10°, (**d**) 30°, (**e**) 20°, (**f**) 10°.

Based on the fundamental understanding of the formation mechanisms of surface residual stress and surface roughness of cerium under diamond cutting, the influence of the rake angle of a cutting tool was investigated. The crystal orientation of the cerium workpiece was Ce(100), and the DOC was 20 Å. Figure 7a–f shows the residual stress distribution of machined surface under different rake angles of a cutting tool, which shows that there were local areas with considerable stress concentration formed on the machined surface. It is found from Figure 7 that the average surface residual stress was larger for the negative rake angle than that for the positive one. Furthermore, the numerical value of surface residual stress decreased with increasing rake angle. It is known that the stress state in the contacting zone between cutting tool and workpiece material was greatly influenced by the rake angle of the cutting tool. With the increase of rake angle from a negative value to positive, the composition of compressive stress gradually decreased. Consequently, both displaced material flow and lattice rotations changed with the rake angle of the cutting tool.

**Figure 8:**
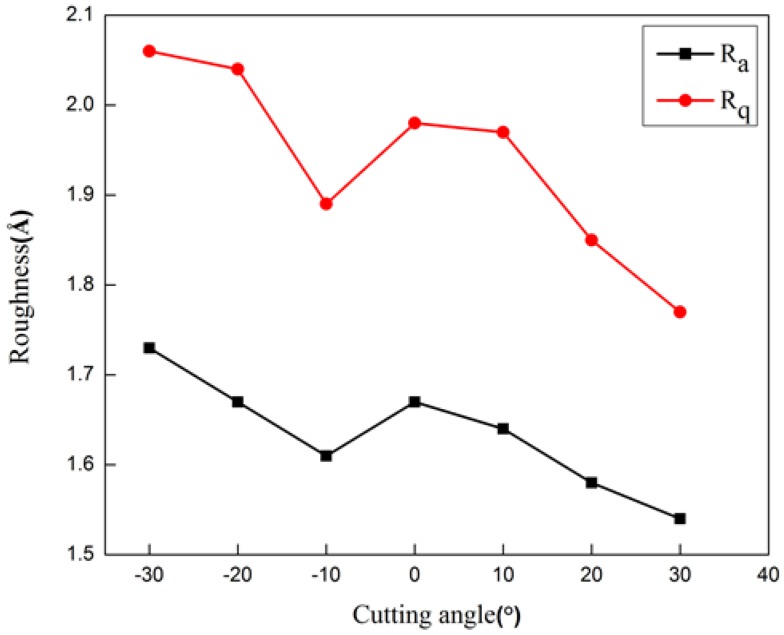
Influence of rake angle on roughness of machined surface.

Figure 8 plots the variations of contour arithmetic mean deviation $R_{a}$ and the mean square deviation $R_{q}$ with rank angle of the cutting tool. The surface roughness obtained under positive rake angle was smaller than that under a negative rake angle. Furthermore, surface roughness decreased with the increase of the rake angle. Figure 8 demonstrates that processing parameters had a more pronounced influence on $R_{q}$ than that on $R_{a}$, indicating that $R_{q}$ was more sensitive to atomic points deviating from the average plane in the evaluation. Therefore, it is indicated that the rake angle of 30° was optimal for the diamond cutting of cerium for achieving minimum residual stress and surface roughness.

### 3.3. Effect of Crystal Orientation

**Figure 9:**
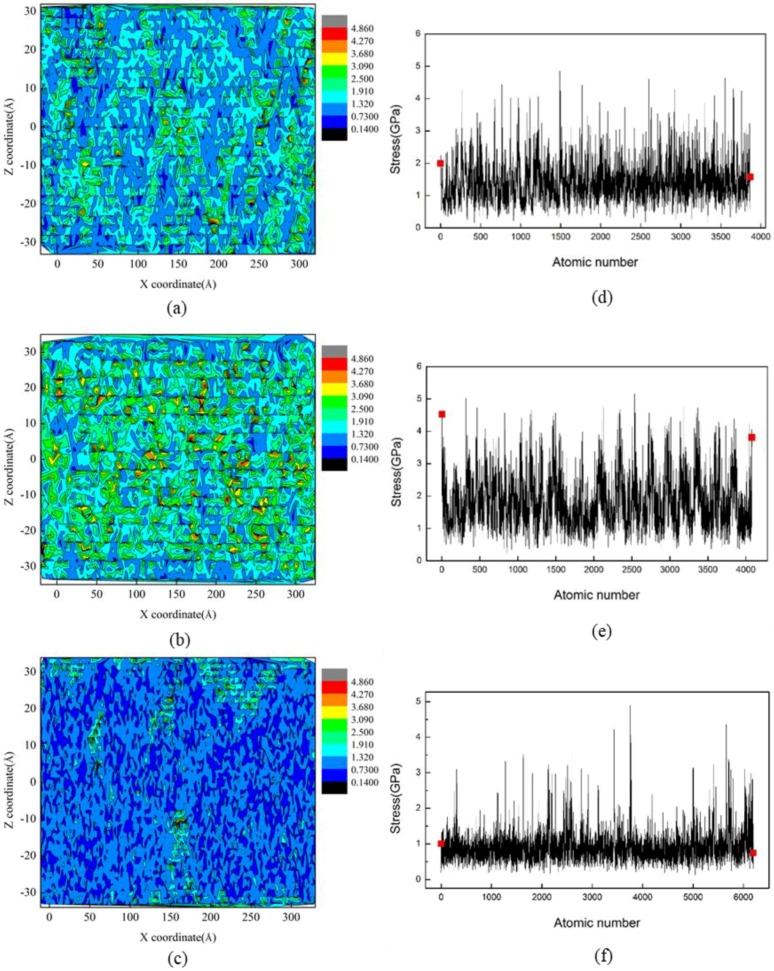
Magnitude and distribution of residual stress. Crystal orientation: (**a**,**d**) Ce(100), (**b**,**e**) Ce(110), (**c**,**f**) Ce(111).

The influence of crystal orientation on the diamond cutting of cerium under the optimal rake angle of 30° was also investigated. Three kinds of single crystal, Ce(110), Ce(111), and Ce(100) were considered, respectively, and all other machining parameters were the same, with a DOC of 20 Å and a rake angle of 30°. Figure 9 shows that both magnitude and distribution of residual stress of surface were different for different crystal orientations. The magnitude of residual stress of Ce(111) was significantly lower than that of Ce(100) and Ce(110). Furthermore, Ce(110) had the most nonuniform distribution due to the most serious dislocation slip-dominated plastic deformation occurring during the cutting process. It is known that the geometry of activated slip systems with respect to a free surface is different for different crystal orientations. Consequently, different intersections between dislocation and free surface lead to different residual stress for different crystal orientations [37,38].

**Figure 10:**
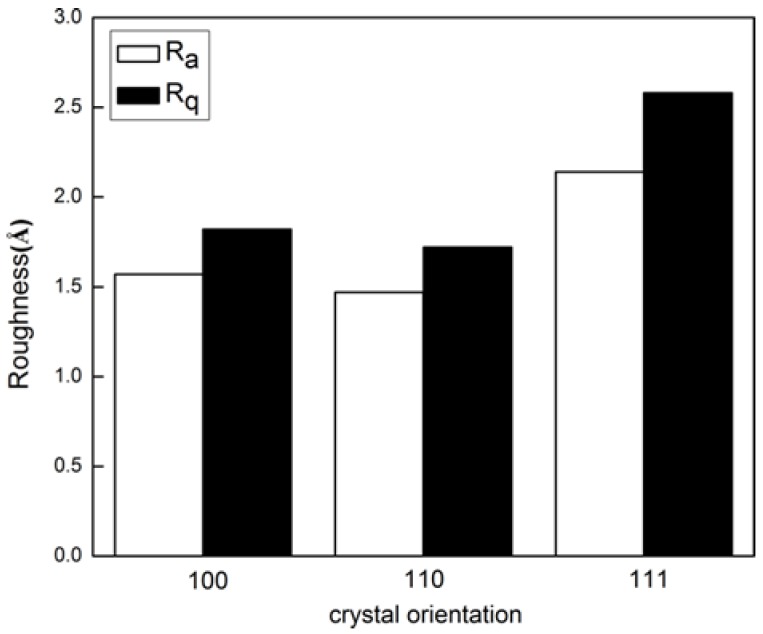
Crystal orientation dependence of surface roughness of cerium.

Figure 10 shows the diagrams of $R_{a}$ and $R_{q}$ of machined surface with different crystal orientations. It is seen from Figure 10 that the surface roughness of Ce(111) was the largest, while the surface roughness value of Ce(110) and Ce(100) was almost the same. It is known that the spacing of crystal planes and the density of covalent bonds vary with crystal orientation. The strength and wear resistance to bond broken of Ce(111) were higher than Ce(110) and Ce(100).Therefore, it is indicated that the Ce(100) crystal orientation was optimal for the diamond cutting of cerium due to low residual stress and moderate roughness.

### 3.4. Effect of DOC

**Figure 11:**
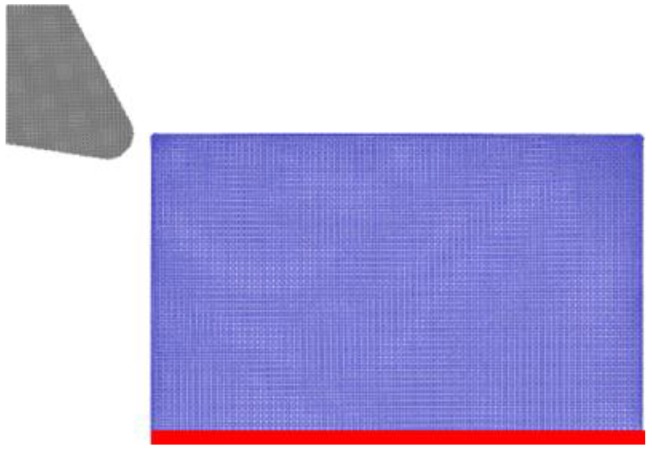
MD simulation model of different cutting depth.

The influence of DOC on the diamond cutting of cerium was further studied under the machining conditions of a rake angle of 30° and a crystal orientation of Ce(100). The material removal mode of plowing and cutting is closely associated with the DOC. The critical DOC at which removal mode transition occurs was dependent on the sharpness of the cutting tool due to stress concentration. Therefore, to magnify the impact of DOC, a blunt cutting edge with an edge radius of 2 nm was utilized, as shown in Figure 11. Five cutting depths, as 2 Å, 6 Å, 10 Å, 15 Å, and 20 Å, were utilized to address the influence of DOC [17].

**Figure 12:**
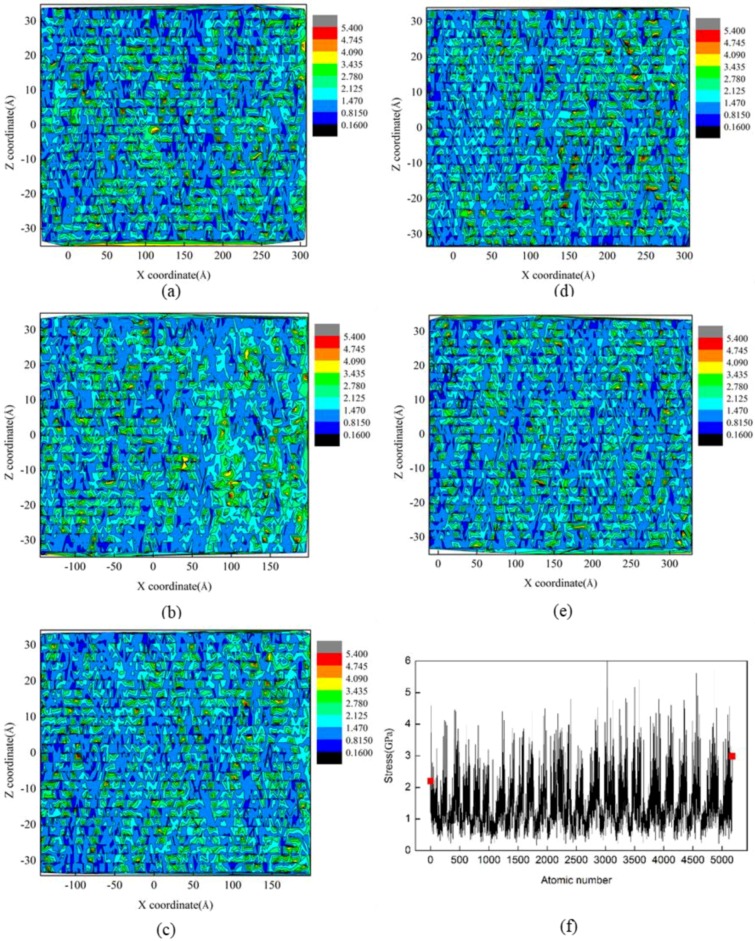
Distribution of residual stress at different DOCs: (**a**) 2 Å, (**b**) 6 Å, (**c**) 10 Å, (**d**) 15 Å, and (**e**) 20 Å. (**f**) Magnitude of residual stress at the DOC of 10 Å.

The DOC had a significant impact on the material removal mode of cerium under the diamond-cutting process. Specifically, material removal can be achieved through plowing or cutting under different DOCs [17]. Son et al. theoretically calculated the minimum cutting depth of chip formation by diamond-cutting tools, which can described in Equation (6) as [39]:

(6) $$h_{min}=R\left(1-\cos\left(\frac{\pi -\beta }{2}\right)\right)$$

where *R* is the edge radius of cutting tool and β is the friction angle. Simulation results show that there was chip formation under the cutting mode using a DOC of 15 Å and 20 Å, while displaced material mainly accumulated on both sides of the formed groove under the plowing mode under a DOC of 2 Å, 6 Å, and 10 Å [17]. It is found from Figure 12 that the DOC had a nontrivial size effect on the residual stress of the machined surface. The residual stress distribution of the machined surface was also extremely uneven. In particular, the DOC of 10 Å had obvious stress concentration near the cutting edge of the workpiece.

**Figure 13:**
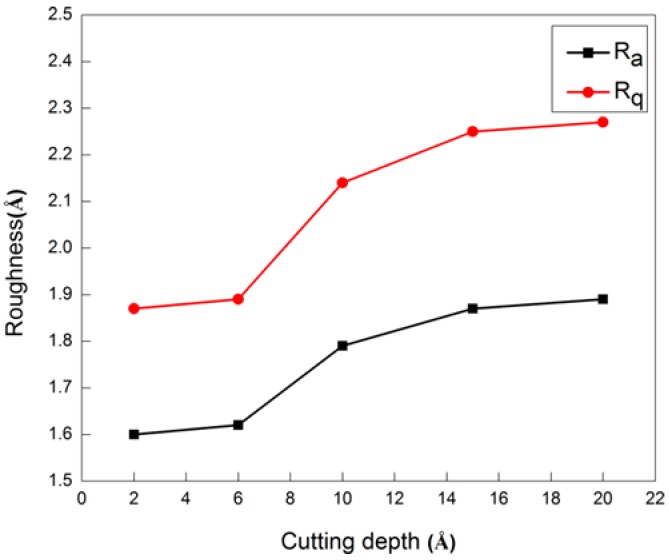
Influence of depth of cut (DOC) on surface roughness.

Figure 13 shows that a small DOC of 2 Å or 6 Å led to small roughness of $R_{a}$ or $R_{q}$. With the increase of DOC, the variation of roughness was relatively smooth, especially after the chip formation. The result indicates that the DOC was less sensitive than other processing parameters in the affecting of surface roughness.

## 4. Summary

In summary, we performed MD modeling and simulation to elucidate the formation mechanisms of surface residual stress and surface roughness of single crystalline cerium under ultraprecision diamond cutting. Simulation results revealed that surface roughness is closely correlated with a dislocation slip that leads to the formation of surface steps on a machined surface. The formation of residual stress after cutting processing is determined by both lattice distortion and dislocation glide. It was found that machining parameters, such as DOC, rake angle of the cutting tool, and crystal orientation of the workpiece have a strong influence on residual stress and surface roughness. The optimal machining conditions, i.e., a rake angle of 30°, with a shallow DOC, and a crystal orientation of Ce(100), can lead to high quality of machined surface of cerium under diamond cutting.
